# Effects of microvibration stimulation on developmental potential of discarded germinal vesicle oocytes of human

**DOI:** 10.3389/fendo.2022.1028557

**Published:** 2022-10-17

**Authors:** Qinli Liu, Sen Zhao, Jian Zhou, Ping Liu, Bo Huo

**Affiliations:** ^1^ Department of Mechanics, School of Aerospace Engineering, Beijing Institute of Technology, Beijing, China; ^2^ Reproductive Medical Center, Amcare Women’s & Children’s Hospital, Tianjin, China; ^3^ Reproductive Medical Centre, Department of Obstetrics and Gynecology, Peking University Third Hospital, Beijing, China; ^4^ Sports Artificial Intelligence Institute, Capital University of Physical Education and Sports, Beijing, China

**Keywords:** immature oocyte, microvibration, dynamic culture, parthenogenetic development, oocyte activation

## Abstract

**Objective:**

This research aims to study the effects of continuous microvibration stimulation on the parthenogenetic development of human germinal vesicle oocytes.

**Methods:**

Ninety-five discarded germinal vesicle oocytes from intracytoplasmic sperm injection treatment (ICSI) cycles performed at Amcare Women’s & Children’s Hospital between January and December 2021 were used for conventional static culture as well as 10 Hz microvibration culture. We investigated the differences between the two groups in terms of oocyte maturation rate, parthenogenetic activation rate, and parthenogenetic blastocyst formation rate.

**Results:**

The static culture and 10 Hz microvibration culture of 95 oocytes showed that the parthenogenetic blastocyst formation rate in the microvibration culture group was significantly higher than those in the traditional static culture group.

**Conclusion:**

A continuous microvibration stimulation can significantly improve the parthenogenetic developmental potential of human immature oocytes.

## Introduction

Parthenogenetic stem cells are derived from inner cell mass of a parthenogenetic blastocyst have developed from a single MII oocyte without sperm. The major histocompatibility complex (MHC) allele of parthenogenetic embryonic stem cells is homozygous, which can greatly reduce the immune rejection after autologous transplantation (1). In addition, parthenogenetic embryos originate from oocytes and cannot develop into individuals, the study of parthenogenetic embryonic stem cells avoids many ethical problems. At the same time, parthenogenetic embryos can be used as a useful tool to study the expression and function of imprinted genes, and provide cell source for regenerative mediclne. Approximately 15%-20% of immature oocytes are discarded following intracytoplasmic sperm injection (ICSI) procedures. It will be very valuable to obtain parthenogenetic embryonic stem cells by parthenogenetic activation after *in vitro* maturation of these immature oocytes (2).

*In vitro* maturation (IVM) of immature oocytes refers to the maturation of immature oocytes obtained in *in vitro* culture. To date, more than 5000 IVM babies have been born worldwide (3), but their clinical pregnancy rate remains low, at approximately 30-40% (3, 4). The current IVM technique suffers from drawbacks, such as (1) low IVM rate, (2) unsynchronized maturation of the nucleus and cytoplasm in mature oocytes, and (3) low developmental potential of the formed embryos (5, 6). These problems indicate that the IVM technique is in urgent need of optimization.

The female ovary and fallopian tubes are important sites for follicular growth, egg transport, fertilization, and early embryonic development (7). Besides the development of assisted reproductive technologies and the understanding of the delicate and complex physiological functions of the fallopian tubes, the physical environment of the fallopian tubes has also received increasing attention from researchers (8, 9). The migration of eggs and embryos through the fallopian tubes is influenced by a combination of tubal patency, structural integrity of the mucosal epithelium and ciliary vibrations, tubal fluid flow direction, and muscle contractile function and neural regulation (10). The mechanical effect of the function of the fallopian tube in transporting eggs and embryos is reflected in the systematic production of forces by the muscles, cilia, and tubal secretory cells, which move or fix the eggs and embryos (7, 11).

With the development of human-assisted reproduction techniques, the current culture of oocytes and embryos is dominated by static culture. Current research in improving *in vitro* systems focuses on chemical factors, including changes in organic salt composition, energy substrate concentrations, and amino acid composition, and various growth factors or reduction in the number of other substances. However, in the process of constantly seeking to improve *in vitro* culture conditions, not only the requirements of chemical factors of oocytes and embryos need to be considered, but also the potential physical environment may also be an important influencing factor. Oocytes and embryos are exposed to not only altered chemistry during their passage through the female reproductive tract but also mild mechanical stimuli (11–13). In contrast, relatively less research has been done on the physical environment in which preimplantation embryos are placed and the role of culture platforms or equipment in influencing embryonic development in the laboratory. The stimulatory effects of vibration on living systems are well known and play an important role in mechanotransduction, which is necessary for the survival of cells and higher organisms. Early human embryos are exposed *in vivo* to cilia vibrations at 5-20 Hz (13, 14). Isachenko et al. found that exposure of early embryos in a microvibration culture to 56 Hz vibrations significantly increased the rate of human embryo implantation (Isachenko, et al., 2017). The research by Mizobe et al. showed that applying microvibration stimulation to immature oocytes of pigs significantly improved the parthenogenetic developmental potential of immature oocytes (15).

All these studies demonstrated that mammalian oocytes and early embryos can sense microvibrational stimulation, which further enhances embryonic developmental potential. However, fewer studies investigated the mechanical stimulation of human immature oocytes. Currently, the culture of human immature oocytes in the IVM technique is mainly static. Whether the lack of a mechanical environment is one of the reasons for the low efficiency of human immature oocytes in *in vitro* culture remains unclear. Therefore, the main objective of this study was to investigate whether microvibration stimulation could improve the IVM rate and embryonic developmental potential of human immature oocytes.

## Materials and methods

### Acquisition of immature oocytes

In this study, infertile patients who underwent *in vitro* fertilization (IVF) treatment at the Reproductive Medicine Center of the Amcare Women’s & Children’s Hospital between January and December 2021 were examined under the premise of approval by the ethical committee of Amcare Women’s & Children’s Hospital. Female patients less than 35 years old were selected. After patient couples had signed an informed consent form, 95 immature oocytes voluntarily donated were used for this study. Agonist or antagonist protocols were applied as routine ovulation stimulation programs. The stimulation protocol was based on the patient’s age, history of ovulation, history of pelvic surgery, basal follicle-stimulating hormone (FSH) level, and other considerations. After pituitary suppression was achieved *via* a gonadotrophin-releasing hormone antagonist or agonist, ovarian stimulation was performed using FSH. The dose was adjusted based on the individual response. The follicle number and size were monitored *via* ovarian ultrasonography, and serum 17β-estradiol levels were measured after 3, 4, or 5 days of gonadotropin treatment and then as needed until retrieval. Human chorionic gonadotropin (hCG) was administered when three or more follicles ≥16 mm were present. Oocytes were retrieved by vaginal ultrasound-guided follicular aspiration 36–37 h after hCG administration. During the ICSI cycle, some cumulus cells were removed 4 h after egg retrieval. One to two layers of granulosa cells were preserved, and oocyte maturation was observed under an inverted microscope. The germinal-vesicle stage (GV) oocytes were cultured for maturation.

### IVM of immature oocytes

GV-stage oocytes were placed in a Kitazato IVM culture medium (Kitazato Cat.# SK-IVMC-50, Japan) supplemented with 0.075 IU FSH and 0.075 IU LH and incubated for 24 h to observe maturation. Oocyte maturation was determined by excluding the first polar body.

### Vibrational culture of oocytes and embryos

**Figure 1:**
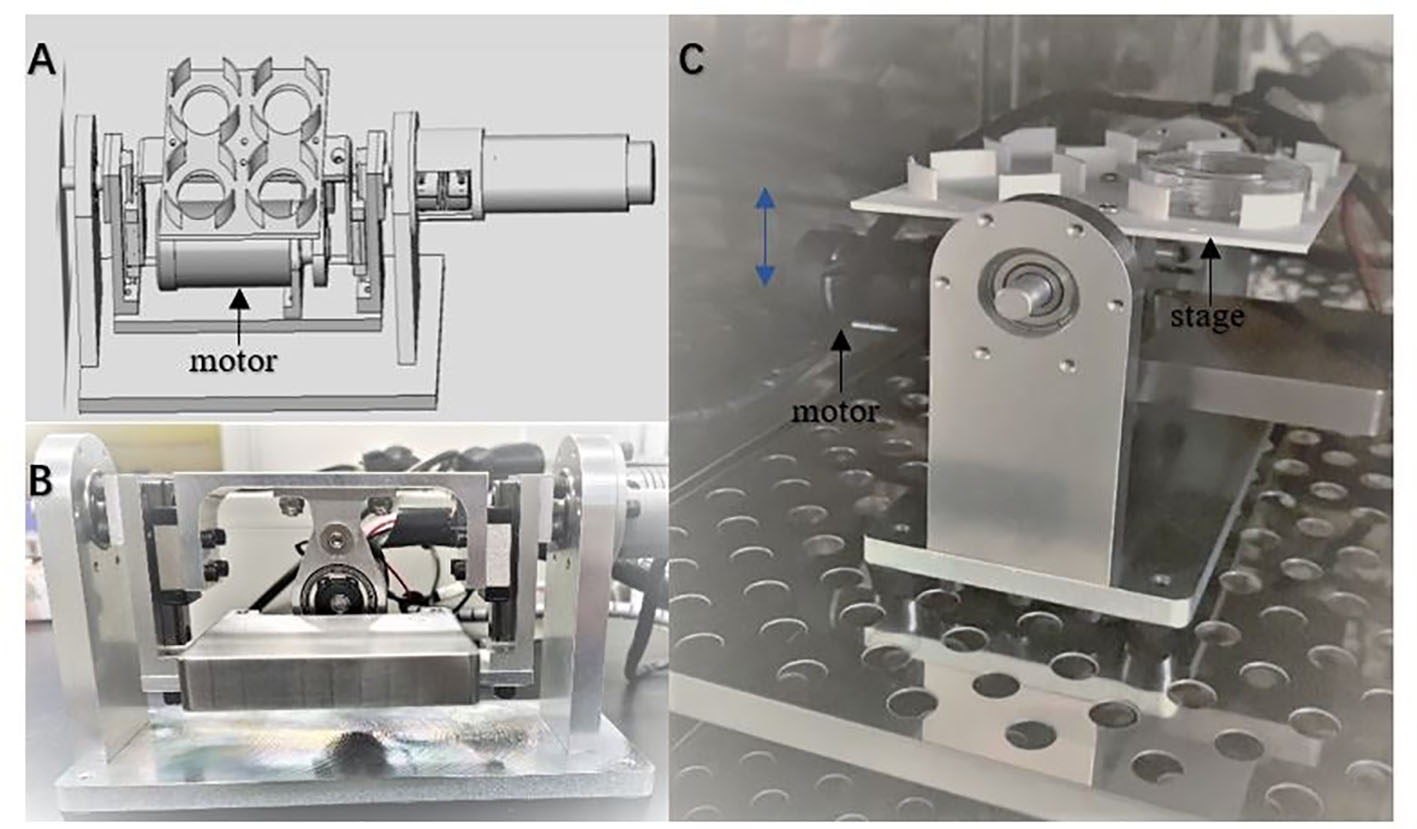
The custom-made device providing vibration condition for cell culture. The device can be used in a standard humidified incubator with a waterproof unit **(A)**. The schematic diagram of the device **(B, C)**. The photos of the device from frontal and side view. Culture dishes are set on the stage. The blue double arrow indicates the movement direction of the stage driven by the motor.

A custom-made vibration device (**Figure 1**) was used for oocytes and embryos cultured, which provided continuous vibration stimulation for oocytes and embryos by simulating the up and down beating of cilia at a vibration frequency of 10 Hz. There is a stage in the device supporting petri dish. The dish and the stage vibrate up and down with the motor, the loading mode of vibration is set as continuous loading with 10 Hz in vibration.

### Parthenogenetic activation

Ionomycin activation dish was prepared by adding G-IVF PLUS to an aliquot of ionomycin (final concentration of 5μM) 30 min before oocyte activation. Drops (50 μL per drop) were formed, covered with 3 mL of OVOIL, and equilibrated in an incubator. Then, 6-dimethylaminoputine (6-DMAP), activation dish was prepared by adding G-IVF PLUS (fertilization culture medium, Vitrolife) to an aliquot of 6-DMAP (final concentration of 1mM). Drops (50 μL per drop) were formed, covered with 3 mL of OVOIL, and equilibrated in an incubator. In addition, three G-IVF PLUS dishes were made for the oocyte wash. G-1 PLUS (cleavage embryo culture medium, Vitrolife) dishes were prepared for culture oocytes after oocyte activation. The oocytes were washed two to three times to ionomycin dishes and then incubated in the incubator for 5 min. After being washed in G-IVF PLUS dishes, the oocytes were transferred into 6-DMAP microdroplets and washed twice or three times. After incubation in the incubator for 4 h, the oocytes were washed in G-IVF PLUS dishes and cultured in G-1 PLUS dishes.

### Embryo culture

The oocytes from the parthenogenetic activation treatment were transferred into G1 PLUS. Cleavage after parthenogenetic activation is considered oocyte activated. Activated oocytes were transferred to G2 PLUS (blastocyst culture medium, Vitrolife) on the third day, and the development of parthenogenetic embryos in each group was observed and recorded daily until the sixth day. The development of parthenogenetic embryos in the dynamic and static culture groups was observed and recorded.

## Statistical methods

In this study, immature oocytes from women aged less than 35 years were selected for the study. SPSS 17.0 software (SPSS Inc. Chicago, IL, USA) was used for statistical analysis, and the *χ* ^2^ test was used to compare the count data. *P* < 0.05 indicated a statistically significant difference.

## Results

### Effect of microvibration on IVM of immature oocytes

IVM of 95 immature oocytes was carried out. 60 oocytes were cultured statically and 35 with microvibration. 38 immature oocytes matured in static culture group and 30 immature oocytes matured in the microvibration culture group, indicating that the oocyte maturation rates were 63.33% and 85.71%, respectively (**Table 1**). The results showed that the maturation rate of oocytes cultured with 10 Hz continuous vibration was higher than that of oocytes in conventional static culture (*P* = 0.033).

**Table 1:** Activation and parthenogenetic embryonic development of oocytes matured *in vitro*.

	Oocytes	Matured to MII	Activated	2-7-cell	≥8-cell	Blastocyst
Static culture group	60	38 (63.33)	32 (84.21)	30 (93.75)	2 (6.25)	0
Vibration culture group	35	30 (85.71)^a^	27 (90.00)	15 (55.56)	12 (44.44)	5^a^

Values are number (percentage); ^a^P<0.05.

### Effect of microvibration stimulation on oocyte activation

After the activation of 38 mature oocytes in the static culture group and 30 mature oocytes in the microvibration culture group, 32 oocytes were activated in the static culture group and 27 oocytes were activated the microvibration group, with activation rates of 84.21% and 90.00%, respectively (**Table1**). No significant difference was found in oocyte activation rates between the two groups. 44.44% of activated oocytes can develop to ≥ 8 cells stage at day 3 in the microvibration culture group, while the rate was only 5.6% in static culture group (**Table 1**). The results showed that the microvibration stimulation can promote early embryo developmental potential (*P* = 0.010).

### Effect of microvibration stimulation on the parthenogenetic development

**Figure 2:**
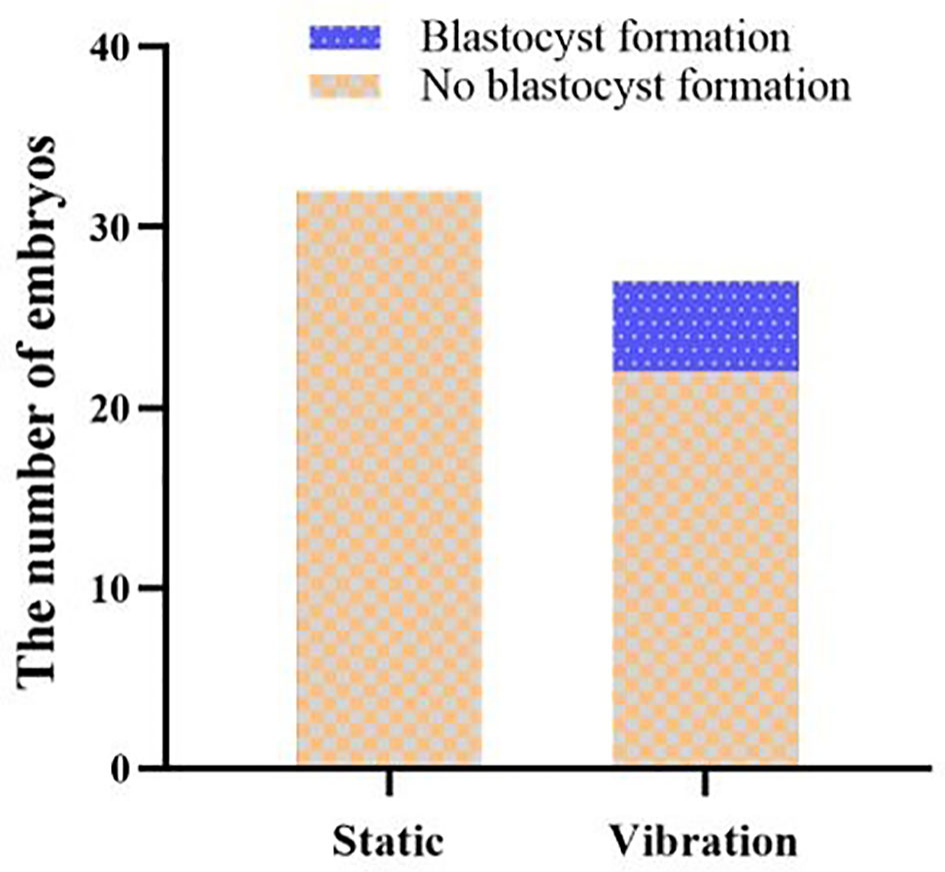
Effect of microvibration stimulation on the parthenogenetic development. The number of embryos forming (blue box) or not forming (orange box) blastocyst during parthenogenesis.

None of the 32 parthenogenetically activated oocytes formed blastocysts in the static culture group, while 5 parthenogenetically activated oocytes developed to blastocysts in the microvibration group (**Figure 2**). The development rates of parthenogenetic blastocysts were 0.00% and 18.52%, respectively. The results showed that the use of 10 Hz constant vibration significantly (*P* = 0.016) increased the rate of parthenogenetic blastocyst formation.

## Discussion

In this study, we found that the IVM of human immature oocytes and the formation rate of parthenogenetic blastocysts could be significantly increased by applying10 Hz continuous vibration to human immature oocytes. The oocyte maturation rate in the static culture group in this study was 63.33%, which was similar to the previously reported results (16, 17). Recent studies have found that the culture of immature eggs with CAPA-IVM culture system (add C−type natriuretic peptide) can significantly increase the developmental potential of human and sheep embryos (18, 19). However, these studies are different from the purpose of our present study. We paid more attention to the mechanical environment of oocytes and embryos, and the effect of mechanical stimulation on embryonic development, in order to mimic the physical environment of early embryos. In fact, the mechanical effect of the function of the fallopian tube in transporting eggs and embryos is reflected in the systematic production of forces by the muscles, cilia, and tubal secretory cells, which move or fix the eggs and embryos (7, 11).

The rate of parthenogenetic blastocyst formation in the static embryo group in this study was 0.00%, which was similar to the previously reported results, both showing low oocyte maturation and low developmental potential (2). In the present study, we used 10 Hz continuous vibration on human immature oocytes. The results showed the developmental potential were significantly improved *in vitro*. This may contribute to the improvement in IVM efficiency and immature oocyte-derived parthenogenic blastocysts.

Mizobe et al. applied vibration at 20 Hz for 5 s per minute to immature oocytes of pigs and found that the parthenogenetic potential significantly increased (15). This was similar to the results of the present study. Several studies also found that microvibration stimulation positively promoted embryonic development of mammals, which was similar to the results of the present study (20–23).

Currently, the main problem of *in vitro* matured oocytes is that the nucleus and cytoplasm of oocytes are not synchronized with each other compared with *in vivo* mature oocytes (5). Mizobe et al. used microvibrational stimulation in culture and found that the cytoplasmic maturation of porcine oocytes could be improved (15). The maturation of oocytes includes both nuclear and cytoplasmic maturation (24), and the cytoskeleton plays an important role in the maturation of oocytes. The cytoskeleton is involved in oocyte spindle migration, oocyte polarization, polar body expulsion, cytoplasmic division, and organelle rearrangement (25, 26). Besides its role in maintaining and changing the morphology of cells and the positioning of organelles, the cytoskeleton can also conduct and sense changes in the mechanical environment in which it is placed. The cytoskeleton can sense changes in the mechanical environment in which the cell is located and transmits mechanical signals from cell to cell, causing changes in cellular life activities. In the present study, 10 Hz vibrational stimulation applied to immature oocytes increased the developmental potential of oocytes. We speculate that this increase in developmental potential might be due to the transmission of mechanical signals through the cytoskeleton to the cell, causing an intracellular response that regulated the maturation of the oocyte, including the maturation of the cytoplasm and the maturation of the nucleus, and thus increased its developmental potential.

Therefore, this study concluded that, besides the role of some chemical factors, the mechanical environment around the cells should also be taken into account when looking for ways to improve the IVM technique so that the growth and developmental environment of the cells *in vivo* are truly simulated. This novel study showed that dynamic culture of human immature oocytes could improve their IVM rate and parthenogenetic embryo developmental potential, which may provided a new technical means to improve the IVM technique and obtain a new method for obtaining parthenogenetic stem cells, i.e. embryonic stem cells of immature oocyte origin. In order to reduce the impact of individual differences, we analyzed the development of sister oocytes (at least two immature oocytes come from the same patient in the same cycle). Among 69 immature oocytes, 23 immature oocytes were sister oocytes. We splitted them into two parts, one part was used for dynamic culture and the other part for static culture. The results showed that in the dynamic group, 3 of the 10 activated oocytes developed to parthenogenetic blastocysts. In the static culture group, 7 activated oocytes did not develop into parthenogenetic blastocysts. Due to the limited source of immature oocytes, the limitation of this study is that the sample size is small. We will use animal experiments to verify the results in the following research. Meanwhile, exploring the optimal mechanistic parameters and the molecular mechanisms of mechanistic stimulation will be the subjects of future research.

## Ethic statement

The studies involving human participants were reviewed and approved by Amcare Women’s & Children’s Hospital Ethics Committee for Reproductive Medicine. The patients/participants provided their written informed consent to participate in this study.

## Data availability statement

The original contributions presented in the study are included in the article/supplementary material. Further inquiries can be directed to the corresponding authors.

## Author contributions

BH and QL conceived and designed the study. QL and PL performed the experiments and analyzed the data. QL and BH wrote the manuscript. JZ assisted in writing the manuscript. SZ and QL designed and developed the device. All authors contributed to the article and approved the submitted version.

## Funding

This study was supported by the National Key Research and Development Program Grant (National Natural Science Foundation of China Grant Program No. 12072034).

## Conflict of interest

The authors declare that the research was conducted in the absence of any commercial or financial relationships that could be construed as a potential conflict of interest.

## Publisher’s note

All claims expressed in this article are solely those of the authors and do not necessarily represent those of their affiliated organizations, or those of the publisher, the editors and the reviewers. Any product that may be evaluated in this article, or claim that may be made by its manufacturer, is not guaranteed or endorsed by the publisher.
